# Investigation of DRD2, NRG1 and PI3K/AKT/MTOR expression levels before and after antipsychotic treatment in female patients with schizophrenia

**DOI:** 10.1192/j.eurpsy.2025.344

**Published:** 2025-08-26

**Authors:** O. Sehitogullari, Ç. Ozulu, T. D. Berkol, A. E. Bayrak

**Affiliations:** 1 Psychiatry, Health Sciences University Bakirkoy Prof. Dr. Mazhar Osman Mental Health and Neurological Diseases Training and Research Hospital, Istanbul; 2 Moleculer Biology and Genetics, Istanbul University, Istanbul; 3 Medical Genetics, Istanbul University, Istanbul Faculty of Medicine, Istanbul, Türkiye

## Abstract

**Introduction:**

Schizophrenia, affecting about 1% of the global population, is marked by positive, negative, and cognitive symptoms, leading to significant disability and a shortened lifespan due to physical health issues. Early intervention is crucial, as untreated psychosis can persist. This study examines changes in mRNA expression in the NRG-1/PI3K-AKT and DRD2 pathways in treated and untreated patients, hypothesizing that expression decreases and varies with treatment.

**Objectives:**

We aim to assess the impact of antipsychotic treatment on the expression levels of the PI3K/AKT/m-TOR signaling pathway, linked to the neurodevelopmental hypothesis of schizophrenia, and associated with the dopaminergic DRD-2 and glutamatergic NRG-1 systems. This study will examine mRNA expression of DRD-2, NRG-1, and PI3K/AKT/m-TOR in leukocytes from female schizophrenia patients before and after treatment. The findings may enhance our understanding of schizophrenia’s pathogenesis, identify potential genetic markers, and clarify the molecular effects of antipsychotic drugs.

**Methods:**

Our study includes 25 healthy female volunteers and 25 female schizophrenia inpatients from Bakırkoy Prof. Dr. Mazhar Osman Mental Health and Neurological Diseases Training and Research Hospital. These inpatients met the experiment criteria, had not received treatment in the last 3 months, and were diagnosed with schizophrenia spectrum disorder according to DSM V criteria. After obtaining written consent, a sociodemographic form was completed. The Positive and Negative Symptom Scale (PANSS) was administered to eligible patients. Blood samples for DRD-2, NRG-1, and PI3K/AKT-1/mTOR mRNA expression analysis were collected from patients with PANSS scores of 95 or above before treatment and after scores decreased to 58 or below. Expression levels were determined by RT-PCR.

**Results:**

In untreated schizophrenia patients, NRG-1 mRNA expression was significantly lower, while PI3K and mTOR mRNA expressions were significantly higher compared to the healthy control group, with no change in AKT-1 mRNA expression as shown in **Table 1**. Compared to healthy controls, treated patients showed increased NRG-1 mRNA, unchanged PI3K and AKT-1 mRNA, and decreased mTOR mRNA as shown in **Table 2**. In treated patients, NRG-1 mRNA expression was significantly higher, and PI3K and mTOR mRNA expressions were significantly lower compared to untreated patients, with no change in AKT-1 as shown in **Table 3**. DRD-2 mRNA expression was undetectable in all groups.

**Image 1:**

**Figure:**
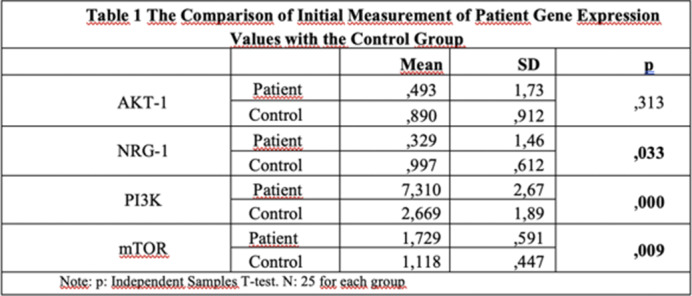

**Image 2:**

**Figure:**
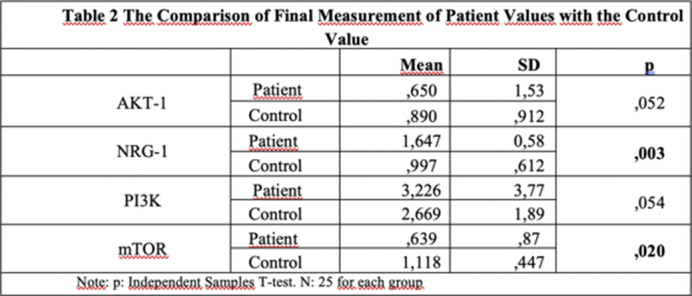

**Image 3:**

**Figure:**
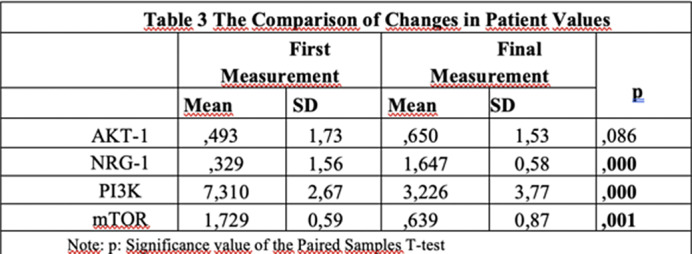

**Conclusions:**

NRG-1, PI3K, and mTOR may contribute to schizophrenia pathogenesis, with NRG-1 and mTOR serving as potential genetic biomarkers. Antipsychotics affect molecular pathways, but not AKT-1, and DRD-2 is not expressed in immune cells.

**Disclosure of Interest:**

None Declared

